# Cross-cultural adaptation and psychometric evaluation of the Brazilian Portuguese version of the childbirth experience questionnaire

**DOI:** 10.1186/s12884-020-03163-9

**Published:** 2020-08-20

**Authors:** Renata Cristina Martins da Silva Vieira, Cristine Homsi Jorge Ferreira, Ricardo de Carvalho Cavalli, Maiara Lazaretti Rodrigues do Prado, Ana Carolina Sartorato Beleza, Patricia Driusso

**Affiliations:** 1 Department of Physical Therapy, Women’s Health Research Laboratory, Federal University of São Carlos (UFSCar), São Carlos, Brazil; 2 Department of Health Sciences, Ribeirão Preto Medical School, University of São Paulo (USP), Av. Bandeirantes, Monte Alegre, Ribeirao Preto, SP Brazil; 3 Department of Gynecology and Obstetrics, Faculty of Medicine, University of São Paulo (USP), São Carlos, Brazil

**Keywords:** Childbirth experience questionnaire, Postpartum, Childbirth satisfaction, Instrument validity

## Abstract

**Background:**

The Childbirth Experience Questionnaire (CEQ) is a tool designed to assess women’s perceptions about labor and delivery. The aim of this study was to perform the cross-cultural adaptation and validation of the Brazilian Portuguese version of the CEQ (CEQ-Br).

**Methods:**

The original version of the CEQ was translated into Portuguese, analyzed by a committee of experts, back translated, and finally submitted to pilot-test. Two applications of the CEQ-Br were performed along with the quality of life questionnaire Medical Outcomes Study 36 - Item Short-Form Health Survey (SF-36). The SPSS software was used for statistical analysis, the intraclass correlation coefficient was used to investigate test-retest reliability, the internal consistency was investigated with the Cronbach’s Alpha, and the construct validity was investigated via the Spearman correlation test. The level of significance was set at 5%.

**Results:**

The study included 308 women with a mean age of 31.1 ± 8.7 years. The internal consistency results for the total CEQ-Br score was considered adequate (0.89), the test-retest showed a substantial result with an ICC of 0.90, and the construct validity was analyzed via the Spearman correlation between all SF-36 dimensions and the total CEQ-B score, the analyses were considered adequate.

**Conclusions:**

The results presented in this CEQ-Br validation study showed that the instrument was reliable in measuring the established psychometric properties and was considered valid. Therefore, the CEQ-Br can be applied to the Brazilian population.

## Background

Researchers and health professionals working with pregnant have shown great interest in understanding women’s perceptions of their experience of childbirth and postpartum. The childbirth experience has often been correlated with both positive and negative health outcomes in postpartum women [1]. Many factors can contribute to the childbirth experience including perceived safety, participation in childbirth, pain experience, family support, obstetric care, experience of previous births, intrapartum analgesia, the pregnant woman’s knowledge of the physiology of labor and delivery, and their involvement in decision making [1–3].

Negative childbirth experiences of childbirth may give rise to feelings of maternal distress, postpartum depression, and even posttraumatic stress disorder, which may compromise subsequent pregnancies and mother-infant interactions, decreasing breastfeeding rates, and affecting the psychomotor development of the child [1–3].

Given the need to assess women’s experience of labor and delivery, Dencker et al. (2010) [3] developed and validated the Childbirth Experience Questionnaire (CEQ) in Swedish. The CEQ is a multidimensional instrument with four dimensions: own capacity, professional support, perceived safety, and participation in labor and delivery. This instrument was subsequently validated in Spain [2], the United Kingdom [4], China [5], and Iran [6]. In all studies, the instrument was considered valid and reproducible, as seen in Table 1.

**Table 1:** Characteristics of CEQ validations carried out in Sweden, the United Kingdom, Spain, China, and Iran

Author Year Country	Participants/ Postpartum Period	Reliability	Validity	Internal consistency
Dencker et al. 2010 [3] Sweden	920 primiparous women with vaginal delivery, emergency cesarean section, instrumental assistance and oxytocin during labor. Up to 1 month postpartum	Good	Good	Own Capacity: 0.82 Professional Support: 0.88 Perceived Safety: 0.78 Participation: 0.62
Walker et al. 2015 [4] United Kingdom	350 primiparous women who had vaginal birth One month postpartum	Substantial	Substantial	Cronbach’s Alpha: 0.90 Own Capacity: 0.79 Professional Support: 0.94 Perceived Safety: 0.94 Participation: 0.72
Soriano-Vidal et al. 2016 [2] Spain	364 primiparous and multiparous women who had vaginal delivery with or without the aid of instruments One to 3 months postpartum	Good	Good	Cronbach’s Alpha: 0.88 Own Capacity: 0.80 Professional Support: 0.90 Perceived Safety: 0.76 Participation: 0.68
Zhu et al. 2019 [5] China	1747 primiparous and multiparous women who had spontaneous vaginal delivery Two to 3 days postpartum	Good/ satisfactory	Good	Cronbach’s Alpha: 0.88
Abbaspoor et al. 2019 [6] Iran	203 women from two different hospitals with spontaneous vaginal births From first to 40 days postpartum	Good	Good	Cronbach’s Alpha: 0.82 Own Capacity: 0.71 Professional Support: 0.78 Perceived Safety: 0.69 Participation: 0.58

Reliability: The extent to which scores for patients who have not changed are the same for repeated measurement under several conditions

Validity: The degree to which an instrument measures the construct(s) it purports to measure and

Internal consistency: The degree of the interrelatedness among the items [7]

In Brazil, there are no validated instruments to assess women’s experience of childbirth and the use of a validated questionnaire seems essential for planning and implementing care strategies that can contribute to a positive childbirth experience for Brazilian women. The aim of this study was to perform the cultural adaptation and validation of the Brazilian Portuguese version of the CEQ (CEQ-Br).

## Method

### Participants, inclusion and exclusion criteria

This is a methodological validity study. The participants were recruited from all over Brazil through social media, from May to December 2017.

The eligibility criteria to participate were: to be over 18 years old and able to read and write in Portuguese, to have had vaginal delivery in the last month following single usual-risk, full-term pregnancy (37 to 42 weeks of gestation) and with no maternal or neonatal diseases. The sample size of this study followed the recommendation of ten times the number of questions of the instrument being validated [7].

The study was approved by the Research Ethics Committee of the Federal University of São Carlos, registration number 1406843. All participants agreed to participate in the study after reading the Informed Consent Form.

### Instrument

The participants answered to the CEQ-Br and the Medical Outcomes Study 36 – Item Short-Form Health Survey (SF-36).

The CEQ questionnaire contained 22 items addressing the experience with the first childbirth, of which 19 questions contained multiple choice and predetermined answers (totally agree, mostly agree, mostly disagree, totally disagree). The other three questions were assessed using the Visual Analog Scale (VAS) [3]. The questionnaire addresses items that assessed the following domains: Own capacity (sense of control, personal feelings during childbirth, and labor pain); Professional support (the perception of care by the obstetric team and the provision of information about childbirth); Perceived safety (sense of security and memories around childbirth); and Participation (possibility of influencing position, movement, and pain relief during labor).

For scoring, items with predetermined answers generated the following values: strongly agree - 4, mostly agree - 3, mostly disagree - 2, strongly disagree − 1. However, the scoring for questions with negative word statements, corresponding to questions 3, 5, 8, 9, and 20, were reversed. For items using the VAS, the scores were converted as follows: 0–40 = 1, 41–60 = 2, 61–80 = 3, and 81–100 = 4 [3].

For the calculation of the final score for the CEQ, items were aggregated to scale scores by adding up the coded values of items in each domain and dividing it by the number of items in this dimension (average). The score ranges from 1 to 4, where higher ratings reflected more positive childbirth experiences and lower scores reflected poorer experiences [3].

CEQ scores were analyzed by domains, and higher values showed better delivery experience, while lower values showed worse experience. Table 2 describes the items of the questionnaire, as well as the need to reverse the score for the indicated questions, according to Dencker et al. 2010 [3].

**Table 2:** Items Covered in the CEQ and Reverse scoring

Number Item	Item
**Domain: Own Capacity (8 items)**	
1	Labor and birth went as I expected.
2	I felt strong during labor and birth.
4	I felt capable during labor and birth.
5^a^	I was tired during labor and birth.
6	I felt happy during labor and birth.
19	I felt that I handled the situation well.
20^a^	As a whole, how painful did you feel childbirth was?
21^b^	As a whole, how much control did you feel you had during childbirth?
**Domain: Professional Support (5 items)**	
13	The professional who accompanied my delivery devoted enough time to me.
14	The professional who accompanied my delivery devoted enough time to my partner.
15	The professional who accompanied my delivery kept me informed about what was happening during labor and birth.
16	The professional who accompanied my delivery understood my needs.
17	I felt very well cared by the professional who accompanied my delivery.
**Domain: Perceived Security (6 items)**	
3^a^	I felt scared during labor and birth.
7	I have many positive memories from childbirth.
8^a^	I have many negative memories from childbirth.
9^a^	Some of my memories fromo childbirth make me feel depressed.
18	My impression of the team’s medical skills made me feel secure.
22^b^	As a whole, how secure did you feel during childbirth?
**Domain: Participation (3 items)**	
10	I felt I could have a say whether I could be up and about or lie down.
11	I felt I could have a say in deciding my birthing position.
12	I felt I could have a say in the choice of pain relief.

^a^Reverse scoring

^b^Visual Analog Scale

SF-36 [8] assessed quality of life. It is widely used and can be applied to different populations, is licensed through The Medical Outcomes Trust, Health Assessment Lab, QualityMetric Incorporated, and Optum Incorporated, and it was validated in Brazilian Portuguese by Ciconelli et al. 1999 [9]. It is composed of 36 items assessing multiple aspects of quality of life in eight dimensions: physical functioning, role-physical, bodily pain, general health, vitality, social functioning, role-emotional, and mental health. It has a final score that can vary from 0 to 100, where zero corresponds to the worst general health and 100 to the best general health.

### Translation

The process of translation and cultural adaptation of the CEQ-Br followed the COnsensus-based Standards for the selection of health Measurement Instruments - COSMIN [10, 11]. Firstly, authorization was requested from the author for the translation and validation of the Brazilian Portuguese version of the CEQ. Two translators fluent in the English language translated the instrument into Brazilian Portuguese. The translations were then reviewed by an expert committee composed of six people who met twice and agreed on the final version of the instrument. Subsequently, the questionnaire was sent to two different English-speaking translators for back-translation. Finally, a pilot test was performed face-to-face with 20 women to determine the instrument’s clarity and coherence. There was no need to change any questions in the questionnaire.

### Procedures

Data collection was carried out online from May to December 2017. The participants accessed a website and completed an evaluation form. The form contained the following items: personal and sociodemographic data (age, education, race, marital status, housing, etc.) and obstetric data (number of children, last childbirth, place of birth, financial assistance for childbirth, professional who attended childbirth, beginning of labor, medication used, induction of labor, interventions during labor, analgesia, duration of the second stage of labor, position taken during the expulsion period, pain during labor).

After completing the assessment form, participants answered the CEQ-Br instrument and the SF-36 twice, the first time within 6 months of delivery and the second time 7 to 10 days after the first time answering it. Finally, the women reported the level of difficulty in answering the CEQ-Br (very easy, easy, neither easy nor difficult, difficult, very difficult) [12]. The participants also informed the time it took to answer the questionnaire.

### Data analysis

For the statistical analysis of the CEQ-Br, the following items and parameters were considered:

1. Internal consistency was calculated using Cronbach’s alpha. Values greater than 0.70 were considered adequate [13].
2. Reliability was analyzed through test-retest using the Intraclass Correlation Coefficient (ICC) test. ICC between 0.40 and 0.75 represents moderate reliability, between 0.75 and 0.90 represents substantial reliability, and greater than 0.90 represents excellent reliability [14].
3. Construct Validity was analyzed by calculating the Spearman correlation coefficient against the scores of each SF-36 dimension (physical functioning, role-physical, bodily pain, general health, vitality, social functioning, role-emotional, and mental health). Correlations with *r* ≥ 0.30 to 0.60 were considered moderate, and correlations with *r* ≥ 0.60 were considered good [13, 14].
4. The measurement error was calculated by dividing the standard deviation of the means by the differences by the square root of 2 [14].
5. Floor and ceiling effects was determined by 15% threshold for patients achieving the highest and lowest score to define a ceiling and floor effect, respectively [15]
6. A significance level of 5% was adopted.

## Results

A total of 320 eligible women were identified through records in health centers and from them, 308 primiparous women, during postpartum period, answered the instruments CEQ-Br, and SF-36 once. One hundred and four women agreed to answer the CEQ-Br twice (test-retest with 10 days between 1st and 2nd CEQ-Br answers). Reasons for declining participation were: uninterested to participate (*n* = 20), caring for the baby hindered (*n* = 158), or lack of time (*n* = 42).

Characteristics of study participants were shown in Table 3. The mean age of participants in this study was 31.1 ± 8.7 years, most women are Caucasian (68%), primiparous (71.1%), and underwent spontaneous vaginal delivery (89.9%), without instrumented delivery (96.1%).

**Table 3:** Characteristics of Participants

Variables	*n* (%)
**Education Level**	
Primary Education	11 (3.5%)
Secondary Education	66 (21.4%)
Tertiary Education	231 (75%)
**Race**	
Asian	8 (25.9%)
Caucasian	212 (68%)
Indigenous	2 (6.4%)
Do not wish to declare	7 (2.2%)
Mixed/Black	79 (25.6%)
**Marital status**	
Cohabiting	297 (96.4%)
Not cohabiting	11 (3.5%)
**Number of children**	
1	220 (71.4%)
2	67 (21.7%)
3	12 (3.8%)
4	7 (2.2%)
**Local Birth**	
Hospital Birth	285 (92.5%)
Home Birth	23 (7.4%)
**Financial Assistance for Childbirth**	
Public Health Service	131 (42.5%)
Health Insurance	109 (6.1%)
Private	68 (22%)
**Childbirth attendants (**multiple options were allowed)	
Obstetrician	241 (78.2%)
Obstetric nurse	193 (62.6%)
Doula	88 (28.6%)
Midwife	16 (5.2%)
**Onset of Labor**	
Spontaneous	277 (89.9%)
Induced	31 (10%)
**Type of delivery**	
Spontaneous vaginal	296 (96.1%)
Instrumental (Forceps or Vacuum Extractor)	12 (3.9%)
**Nonpharmacological methods**	187 (39.3%)
**Analgesia during childbirth**	
Epidural block	31 (10.1%)
Spinal anesthesia	20 (6.5%)
Block of pudendal nerve	1 (0.3%)
Participant did not know what to answer	18 (5.8%)

Table 4 presented the results obtained in each domain of the CEQ-Br, as well as its total score and internal consistency data. Cronbach’s alpha for the total scale was 0.89, and for the subscales: own capacity 0.76; professional support 0.91; perceived safety 0.83; and for participation 0.69. These results demonstrated that internal consistency was high or very high for all domains, therefore the CEQ-Br can be considered an adequate questionnaire to evaluate the experience of childbirth in Brazilian women.

**Table 4:** Internal Consistency and reliability of CEQ-Br domains

Domain	Score results in each domain (mean and standard deviation)	Internal Consistency (Cronbach’s Alpha)	Test-retest (ICC)	Margin of error
Own Capacity	23.6 (± 4.4)	0.76*	0.82	5.5%
Professional Support	17 (±4.2)	0.91*	0.89	4.9%
Perceived Safety	19.7 (±4)	0.83*	0.86	5.1%
Participation	6.7 (±3.5)	0.69*	0.72	4.8%
Total score	66.9 (±10.6)	0.89*	0.90	5.2%
**Results**		**High**	**Substantial**	**Good**

* all correlations were statistically significant *p* < 0.05

Table 5 described the construct validity analyzed through the relationship between the CEQ-Br and SF-36 instruments. The results presented are the total scores of this relationship in each dimension of the SF-36 questionnaire.

**Table 5:** CEQ-Br Construct Validity in relation to the SF-36

SF-36 Domain	CEQ-B
Physical functioning	0.68
Role-physical	0.73
Bodily pain	0.73
General health	0.77
Vitality	0.68
Social functioning	0.79
Role-emotional	0.82
Mental health	0.70

In this study, there was no ceiling and floor effect for the CEQ-Br total score analysis. The participants rated their level of difficulty in answering the questionnaire and the results were: 97 participants (31.5%) rated it as easy, 43 participants (14%) rated it as very easy, 124 (40.3%) neither easy nor difficult, 36 (11.7%) rated it as difficult, and 8 (2.6%) very difficult. The time required to answer the CEQ-Br was also collected and the results were: less than 5 min (57.6%), 6–10 min (16.8%), 11–15 min (22%), 16–20 min (2.3%), unknown (1.3%).

## Discussion

The current study performed the translation into Brazilian Portuguese, cross-cultural adaptation, and validation of the CEQ-Br and analyzed the following psychometric properties of the instrument: internal consistency (Cronbach’s alpha), test-retest (ICC), construct validity, and margin of error. The values found in the present study are very similar to the results found in other validation studies [2, 4–6, 13].

The reliability performed between the first and second CEQ-Br applications with a 10-day interval by test-retest yielded results considered substantial for all CEQ-Br domains and the margin of error was considered good, showing that the CEQ-Br can be considered a reliable questionnaire in scientific research and clinical practice for the assessment of the experience of childbirth in Brazilian women. For the construct validity of the CEQ-Br, a good correlation was identified between the total CEQ-Br score and all SF-36 domains. The SF-36 is widely used for analyzing construct validity in patient-reported health measurement instruments such as the Brazilian Portuguese versions of the Neck/Bournemouth Questionnaire [16] and the Chronic Liver Disease Questionnaire [17]. Participants reported that the CEQ-Br was easy to answer, and only 14.3% of women considered it difficult or very difficult to answer. Similar results were reported in other CEQ validations [2, 4, 5].

This study followed the COSMIN guideline [10, 11] to perform the translation and cultural adaptation of CEQ-Br. The recommended steps were carefully followed including the initial translation by two translators fluent in English, the synthesis of the translation, the back translation by two other translators fluent in the original language of the instrument, the review of versions, and consensus by a committee of experts. The sample size of this study can be considered good, having the adequate number of participants needed to analyze all proposed measurement properties [13].

A limitation of this study is the lack of a confirmatory factor analysis (CFA), because we needed a larger sample size > 400 participants to perform it [18, 19]. The CFA is an important method to validate the structure of the translated instrument. Psychometric properties, including specificity and sensitivity of the CEQ-Br need to be further explored in future studies. Other limitation of this study, referring participants’ level of education, as most of them had tertiary education, which may not properly represent the general population of Brazil. According to census data from 2016, 51% of the population aged 25 and over had at most completed primary education and only 15.3% of the population had completed tertiary education [20].

The eligibility criteria used in our study were similar to those adopted in the validations of the CEQ developed in the United Kingdom, Spain, and Sweden [2–4], and in the present study, the participants were multiparous and primiparous. The participants of the studies from China [5] and Spain [3] have similar schooling level, the present study, developed in Brazil, included a sample with higher schooling level than the Chinese and Spanish studies. In the Brazilian study sample, the age was similar to that presented by the samples of the studies from the United Kingdom [2], Spain [3] and Sweden [4]. Regarding delivery mode, the sample from the Brazilian study had higher levels of spontaneous delivery when compared to the studies run in the United Kingdom [2], Spain [3], and Iran [6]. The reliability result found for the total score of the CEQ-Br is similar to that found in the study in the United Kingdom [4].

The method used to answer the CEQ-Br was through an online platform, similar to how it was done for the validation of the questionnaires in the United Kingdom [4] and China [5]. Ceiling and floor effects were not found in this study, similar to the reports from the validation studies from Spain [2], the United Kingdom [4], and China [5].

Some specific cultural factors can influence maternal satisfaction. Çalik et al. [20] report that women who had their labor induced and received no pain relief methods or had an episiotomy were less satisfied with their labor. Among the study population, only 39.3 and 16.9% of the participants received pain relief, including pharmacological or nonpharmacological methods, respectively. it is important to note that cesarean section was the most common mode of delivery in Brazil, in the 2014–2016 period, comprehending 56% of all births [21]. Negative perceptions of vaginal delivery, related to the fear of labor pain, and also issues such as the fear of not being able to give birth and low quality of care were the aspects most often cited to justify the preference for caesarean section for Brazilian women [22].

The CEQ domains assess a woman’s own capacity in childbirth and can then measure how strong or how tired she felt in going through childbirth. The questionnaire also assesses the relationship between the team of health professionals and the parturient. The use of validated questionnaires in the country is very important for the health team to understand the women’s birth experience and impact of procedures used during delivery in the postpartum period. Perceived safety, i.e. the feeling of being safe during childbirth, can generate positive or negative memories around that time, which along with fears, can trigger postpartum depression or traumatic stress [1]. The domain Participation assesses women’s decisions around childbirth, and knowledge of all these factors presented from the CEQ domains are of great value, as they imply a reduction in the negative impacts that may result from the experience of childbirth [1–3].

The CEQ-Br is an instrument that assesses the experience of childbirth. Given the importance of evaluating the way women refer to their experience, the translation into Brazilian Portuguese and validation of this instrument are of paramount importance and allow the use of this questionnaire in scientific research, clinical practice, and decision making. Future studies should investigate and to compare different samples of Brazilian women in relation to their childbirth experiences using the CEQ-Br, as well as the impact of new strategies to promote and enhance positive experiences.

## Conclusion

This study performed the translation, adaptation, and validation of the Brazilian Portuguese version of the Childbirth Experience Questionnaire (CEQ-Br). The results showed that the instrument is valid and reliable and can be used in the Brazilian population to evaluate the childbirth experience in both primiparous and multiparous women.

## Data Availability

Authors will make data of this study available upon formal request (Patricia Driusso - pdriusso@ufscar.br). All data generated or analyzed during this study was included in this published article.

## Abbreviations

CEQ: Childbirth Experience Questionnaire
CEQ-B: Childbirth Experience Questionnaire - Brazilian Portuguese version
SF-36: Medical Outcomes Study 36-Item Short-Form Health Survey
ICC: Intraclass correlation coefficient
ICF: Informed Consent Form
VAS: Visual Analog Scale
Self-reported: Patient-reported outcome
